# Effects of mental fatigue on basketball decision-making and visual search behavior

**DOI:** 10.3389/fpsyg.2026.1837100

**Published:** 2026-07-27

**Authors:** Mingyuan Li, Yaqi Zhang, Zihao Zheng

**Affiliations:** 1 Department of Military and Physical Education, Jiangxi Institute of Fashion Technology, Nanchang, China; 2 College of Physical Education, Anhui Normal University Huajin Campus, Wuhu, Anhui, China; 3 School of Management, Anhui University, Hefei, Anhui, China

**Keywords:** basketball, decision-making, eye-tracking, mental fatigue, skill level, visual search

## Abstract

**Objective:**

This study investigated the effects of mental fatigue on basketball decision-making performance and visual search behavior, with particular emphasis on whether these effects vary as a function of skill level.

**Methods:**

A 3 (skill level: high-skill, intermediate, novice) × 2 (fatigue status: fatigued, non-fatigued) between-subjects factorial design was employed. Sixty male university students (mean age ≈ 20.2 years) were classified according to basketball skill level as high-skill, intermediate, or novice. Participants completed basketball decision-making tasks based on game footage, in which they were required to decide whether to shoot, pass, or drive while their eye movements were recorded. Mental fatigue was induced prior to testing through a demanding cognitive task. The primary eye-tracking measures were fixation count, mean fixation duration, and saccade count. In addition, scanpaths and heatmaps were examined to provide qualitative insights into visual-search behavior. Quantitative data were analyzed using separate two-way analyses of variance (ANOVAs).

**Results:**

High-skill players demonstrated faster and more accurate responses than intermediate- and novice-skill participants. They also exhibited shorter fixation durations and fewer saccades, a pattern that may be consistent with more selective and economical visual search. Mental fatigue slowed reaction times overall; however, this effect was most pronounced among novice participants, whereas high-skill players appeared comparatively less affected. Saccade count decreased under fatigue, whereas mean fixation duration remained unchanged, suggesting that fatigue-related effects may have been more evident in gaze-shifting measures than in the fixation-based measure assessed here.

**Conclusion:**

Skill level plays a critical role in shaping both decision-making performance and visual-search behavior in basketball. Although mental fatigue may constrain aspects of visual exploration, more skilled players appeared better able to maintain performance under the present task conditions. These findings suggest that expertise may be associated with reduced vulnerability to fatigue-related performance decline under mentally fatiguing conditions.

## 1 Introduction

Basketball is a dynamic, open-skill sport that requires athletes to continuously integrate information about teammates, opponents, ball possession, and spatial relationships in rapidly changing game situations. They must then make timely situational judgments and select appropriate actions (Bourbousson et al., 2010; Ashford et al., 2021). From an ecological dynamics perspective, this decision-making process emerges from the ongoing interaction between athletes and their environment. It depends on the real-time perception of affordances and the capacity to generate adaptive responses (Araújo et al., 2006). Therefore, when mental fatigue impairs attentional control, information integration, or response selection, it may delay basketball decision-making and reduce adaptive performance (Fortes et al., 2022; Sun et al., 2021).

Mental fatigue is generally defined as a psychophysiological state that develops after prolonged engagement in cognitively demanding tasks. It is characterized by increased subjective fatigue, reduced motivation, and impaired cognitive control (Marcora et al., 2009; Van Cutsem et al., 2017; Pageaux and Lepers, 2018). In sport contexts, mental fatigue has been shown to impair physical performance, sport-specific skill execution, and decision-making. However, its effects may vary according to task type, outcome measure, and sport discipline (Habay et al., 2021). In basketball, existing review evidence indicates that mental fatigue may impair technical performance and decision-making quality (Cao et al., 2022). Because mental fatigue induction is typically accompanied by subjective changes, such as increased perceived fatigue, reduced task engagement, or altered alertness, subjective rating scales are commonly used as manipulation checks. These scales help determine whether cognitive load tasks have produced the expected fatigue response (Hopstaken et al., 2016; Veness et al., 2017; Le Mansec et al., 2018; Schampheleer et al., 2025).

Visual search reflects how athletes acquire and use key information during decision-making and is therefore central to understanding sport-specific decision processes. Eye-tracking studies commonly assess attentional allocation using measures such as fixation count, mean fixation duration, and saccade count. Scan paths and heat maps are also used to visualize the spatial distribution and transitions of gaze within a scene (Kredel et al., 2017; Peysakhovich and Hurter, 2018). In team invasion sports, skilled athletes generally identify tactically relevant areas more quickly and allocate visual attention more selectively. By contrast, less experienced athletes tend to display more dispersed search patterns and are more susceptible to irrelevant information (Mann et al., 2007; Roca et al., 2018; Silva et al., 2022). Basketball-specific studies have similarly shown that high-level athletes are more likely to fixate on information areas directly relevant to offensive and defensive decision-making, whereas novices exhibit gaze patterns that are more dispersed, repetitive, and less organized (Jin et al., 2023; Li et al., 2023). Thus, skill-level differences may be reflected not only in decision outcomes but also in how athletes select, extract, and organize task-relevant information.

Mental fatigue may impair cognitive control processes, including sustained attention, interference inhibition, and response inhibition, thereby reducing athletes’ ability to identify and use key situational cues (Faber et al., 2012; Guo et al., 2018; Gantois et al., 2020). In basketball decision-making tasks, athletes must rapidly select relevant cues, evaluate the situation, and choose an appropriate response. Thus, reduced cognitive control is likely to first emerge as a slowing of the decision-making process (Boksem and Tops, 2008; Hopstaken et al., 2016). Research on the speed–accuracy trade-off further suggests that when individuals must balance response speed and decision quality, mental fatigue may lead them to increase response time to maintain task success (Rozand et al., 2015).

The effect of mental fatigue on decision-making performance may be moderated by skill level. Skilled athletes typically have richer sport-specific experience and more stable situational recognition abilities, which enable them to identify key cues more rapidly and make appropriate decisions (Belling et al., 2015; Moran et al., 2019). When cognitive resources are limited, high-level athletes may therefore be better able to maintain decision-making efficiency than novices. By contrast, novices often require broader information search and longer judgment processes because of their limited experience, making them more vulnerable to response delays under mental fatigue conditions (Nian et al., 2023).

However, the effects of mental fatigue on visual search in sport remain inconclusive. Smith et al. (2016) found that mental fatigue impaired soccer-specific decision-making performance, but its effects on most visual search measures were unclear. By contrast, Fortes et al. (2022) reported that declines in decision-making performance among basketball players under mental fatigue were accompanied by changes in fixation count, suggesting that fatigue may alter how athletes sample information from the visual scene. These inconsistent findings indicate that the effects of fatigue on visual search may depend on the task context and the eye-tracking measures examined. Beyond fixation count and fixation duration, saccades reflect rapid gaze shifts between different information areas. Research on mental fatigue has shown that saccades and their dynamic characteristics are sensitive to fatigue states. Therefore, saccade-related measures may provide additional insight into changes in visual exploration under fatigue (Di Stasi et al., 2013; Bafna and Hansen, 2021).

Based on this rationale, the following hypotheses were tested:

*H1* predicted that participants in the fatigued condition would report higher SSS scores than those in the non-fatigued condition, indicating greater subjective sleepiness or lower alertness.

*H2* predicted that higher skill levels would be associated with better decision-making performance and more economical visual-search behavior, as reflected by higher accuracy, shorter response times, shorter mean fixation durations, and fewer saccades.

*H3* predicted that mental fatigue would primarily prolong response times rather than substantially reduce decision-making accuracy.

*H4* predicted that the fatigue-related increase in response time would be greater among novice participants than among intermediate- and high-skill participants.

*H5* predicted that fatigue-related changes in visual search would be more clearly reflected in saccade count than in fixation count or mean fixation duration.

To test these hypotheses, the present study used a basketball-specific video-based decision-making task combined with eye tracking in high-skill, intermediate, and novice participants. Decision-making performance was assessed using accuracy and response time, whereas visual search behavior was characterized using fixation count, mean fixation duration, and saccade count. Scan paths and heat maps were included as supplementary visualizations of gaze distribution and transition patterns. This design allowed us to examine whether mental fatigue slowed decision responses, altered eye-movement behavior, and whether these effects differed by skill level.

## 2 Materials and methods

### 2.1 Participants

This study used a criterion-based approach to classify participants according to basketball skill level. The sample was not intended to represent a fully random sample of the broader basketball population; rather, participants were recruited and classified according to predefined basketball skill criteria to form three distinguishable experience groups. Sixty male university students (mean age ≈ 20.2 years) were recruited and divided into three basketball skill categories: high-skill (*n* = 20), intermediate (*n* = 20), and novice (*n* = 20).

Individuals in the high-skill group held at least a National Level II athlete certificate and had an average of 8.3 years of systematic basketball training (*SD* = 1.86). The intermediate group comprised regularly trained university team players without national athlete certification, with an average of 6.8 years of training experience (*SD* = 1.40). Novices included students without formal basketball training or recognized skill ratings, with an average training experience of less than 1 year (*M* = 0.58 years, *SD* = 1.73). Descriptive characteristics of the sample, including age and training history, are provided in Supplementary Table S1.

Within each skill category, participants were randomly assigned to either a fatigued or non-fatigued condition. This resulted in 30 participants per fatigue condition, with 10 participants in each skill × fatigue combination, for example, high-skill/fatigue and high-skill/non-fatigued. All participants were right-handed, had normal or corrected-to-normal vision, and reported no physical or psychological disorders. Prior to participation, written informed consent was obtained from every individual.

### 2.2 Experimental design

This study was a controlled laboratory experimental study using a 3 (skill level: high-skill, intermediate, novice) × 2 (fatigue status: fatigued, non-fatigued) between-subjects factorial design. Skill level was treated as a criterion-based between-subjects factor, whereas fatigue status was treated as an experimentally manipulated between-subjects factor. Within each skill category, participants were randomly assigned to either the fatigued or non-fatigued condition.

The dependent measures included decision-making performance, assessed using accuracy and reaction time, and eye-tracking metrics, including fixation count, mean fixation duration, and saccade count. To complement these quantitative indices, scan paths and heat maps were also generated to provide qualitative descriptions of gaze behavior. Participants assigned to the fatigued condition completed a cognitively demanding mental fatigue induction task immediately before the decision-making assessment (see Section 2.5). Participants assigned to the non-fatigued condition rested quietly for the same duration. Ethical approval for the study was granted by the Ethics Committee of Anhui University (Ethics Approval Reference No.: IACUCAHU20240801).

A between-subjects design was chosen over a within-subjects approach for several methodological reasons. First, Stroop-based mental fatigue induction protocols are commonly used to impose sustained cognitive demands, and mental fatigue has been shown to affect subsequent physical, psychomotor, and sport-specific performance (Van Cutsem et al., 2017; Habay et al., 2021). Therefore, a within-subjects design would have required a sufficient washout period to reduce possible residual fatigue effects across sessions. Second, repeated exposure to basketball decision-making video clips could generate learning or task-familiarity effects that might confound fatigue-related performance changes. This concern is particularly relevant because visual search and basketball decision-making are closely related to sport-specific expertise (Jin et al., 2023). Third, previous research has documented considerable interindividual variability in mental fatigue susceptibility and performance responses (Habay et al., 2023). In the present study, random assignment within each skill category was therefore used to distribute such variability across fatigue conditions at the group level. Finally, although crossover designs are common in mental fatigue research, between-subjects or between-groups designs have also been used in related sport-based mental fatigue studies, including studies on exercise intensity decision-making and athlete- or skill-level comparisons (Harris and Bray, 2022; Daneshgar-Pironneau et al., 2025; Wu et al., 2025).

### 2.3 Apparatus

The experimental setup centered on a Lenovo ThinkBook laptop, featuring a 16-inch display with a resolution of 1920 × 1080 pixels. Eye movements were tracked with a Tobii Pro X3-120 remote system (Tobii Technology, Sweden), sampling at 120 Hz. Mounted on the desktop, this eye-tracker allowed participants to move their heads with a reasonable degree of freedom while viewing the screen, providing a stable yet flexible environment for capturing visual-search behavior. The system supported screen-based stimuli without compromising the reliability of gaze recordings.

### 2.4 Materials

#### 2.4.1 Decision-making video stimuli

Decision-making stimuli were drawn from recent Chinese Basketball Association (CBA) game footage to improve ecological validity. Each clip began with the start of an offensive sequence and was paused at a critical decision point. Participants could respond by choosing one of three options: pass, drive, or shoot. An expert panel comprising referees, coaches, and research staff reviewed all candidate clips. Their goal was to ensure that each sequence carried clear tactical meaning and presented a level of difficulty appropriate for the task. After careful vetting, the final set included 60 clips: 20 showing passes, 20 depicting drives, and 20 illustrating shots.

#### 2.4.2 Fatigue-induction task

The mental-fatigue induction protocol was adapted from prolonged Stroop-task paradigms previously used in sport-related mental-fatigue research, including soccer-specific decision-making and cricket-relevant performance studies (Smith et al., 2016; Veness et al., 2017). A 600-trial Stroop color-word task was used to provide a fixed and standardized cognitive load across participants. During the task, participants were required to inhibit the dominant tendency to read the word itself and instead respond to the color of the ink. This procedure was intended to continuously tax sustained attention, conflict monitoring, and inhibitory control before the basketball decision-making task.

#### 2.4.3 Manipulation-check measure

Subjective sleepiness and alertness were assessed using the Stanford Sleepiness Scale (SSS; Hoddes et al., 1973). Scores on the SSS range from 1, indicating an active, vital, and alert state, to 7, indicating extreme sleepiness and imminent sleep onset. In the present study, the SSS was used as a brief manipulation-check measure of subjective state change following the fatigue-induction task. The full set of scale descriptors is provided in Supplementary Table S2.

### 2.5 Fatigue induction protocol

Participants assigned to the fatigued condition completed a 600-trial Stroop task immediately before the main experimental assessment. To assess subjective state changes associated with the fatigue-induction protocol, the SSS was administered twice: once prior to the induction phase and again immediately afterward, or following an equivalent rest period for participants in the non-fatigued condition. In the fatigued group, SSS scores increased substantially following the induction task, and all participants showed an increase of at least three points. No participant was excluded on the basis of the manipulation check. Consequently, the final analyzed sample consisted of all 60 participants, with 10 individuals in each combination of skill level and fatigue status.

### 2.6 Procedure

All testing sessions took place in a quiet laboratory environment under standardized conditions. After providing instructions, participants were seated approximately 60 cm from the display and asked to maintain a stable viewing posture throughout the task. Baseline SSS scores were first recorded before the fatigue-induction or rest phase.

Participants then completed either the fatigue-induction phase or a time-matched rest phase, depending on group assignment. Those in the fatigued condition performed the 600-trial Stroop task, whereas those in the non-fatigued condition remained seated quietly for the same duration. Immediately afterward, the SSS was administered again as a manipulation check.

Eye tracking was then calibrated using a standard nine-point procedure, and calibration was accepted only when the mean gaze error was ≤ 0.5°. Following calibration, participants completed a familiarization session consisting of five practice trials. These practice trials used the same video-based decision-making format as the main task and were intended to ensure that participants understood the instructions, were comfortable with the response keys, and were familiar with the general trial structure. Participants were instructed to watch dynamic offensive scenarios drawn from real basketball game footage and, at the moment the video paused, to select the most appropriate action—pass, drive, or shoot—using the “J,” “K,” and “L” keys, respectively. The mapping of keys to responses was clearly displayed on the screen throughout the practice and main phases. Feedback was not provided during practice, consistent with the main task. After the familiarization session, participants completed 30 basketball decision-making trials presented in randomized order. In each trial, a dynamic offensive scenario was shown and participants responded by selecting the action they considered most appropriate—pass, drive, or shoot—using the same key mappings as in the practice phase. Response accuracy, reaction time, and eye-movement data were recorded automatically during task performance. At the end of the session, participants were debriefed and thanked for their participation.

For descriptive scanpath and heatmap presentation, one representative correct trial was identified within each skill × fatigue subgroup. To do so, the median reaction time was first calculated across all correct trials contributed by all participants in that subgroup. The correct trial whose reaction time was closest to that subgroup median was then selected as the representative example. Applying this procedure separately to each of the six subgroups yielded six representative trials for qualitative visualization.

### 2.7 Data analysis

Trial-level performance measures, including accuracy and reaction time, together with eye tracking metrics such as fixation count, mean fixation duration, and saccade count, were extracted in Tobii Pro Lab and then exported for statistical analysis. All analyses were performed using SPSS 26.0 (IBM Corp., Armonk, NY, USA). Before inferential testing, invalid trials, for example, those affected by data loss due to calibration failure or blink-related artifacts, were removed. Reaction time analyses were conducted using correct trials only. Prior to formal analysis, outlier screening was performed at the participant level for all dependent variables. Participants whose mean score on any variable exceeded ± 3 standard deviations from the corresponding group mean were marked for possible exclusion. In fact, no participant met this criterion, and the entire sample of 60 participants was therefore retained for all subsequent analyses.

Effect sizes are reported for the primary inferential tests. For ANOVA results, partial eta squared (ηp^2^) was calculated as the effect-size index, with values of 0.01, 0.06, and 0.14 interpreted as small, medium, and large effects, respectively (Cohen, 1988). For the independent-samples t-test used to verify the fatigue manipulation, Cohen’s d was reported, with benchmarks of 0.20, 0.50, and 0.80 indicating small, medium, and large effects, respectively. The sample size was informed by an *a priori* power analysis conducted in G*Power (Faul et al., 2009). For a 3 × 2 between-subjects ANOVA with a medium effect size (*f* = 0.25), an alpha level of 0.05, and a target power of 0.80, the minimum required total sample size was approximately 52. To allow for potential data loss and individual variability, 60 participants were recruited, which provided adequate statistical power for detecting medium-to-large effects.

## 3 Results

### 3.1 Stanford sleepiness scale (SSS) scores

All participants in the fatigued condition met the pre-established SSS increase criterion (≥3 points), supporting the effectiveness of the manipulation across skill groups. No participant in the fatigue condition was excluded on this basis. Descriptive statistics were computed for Stanford Sleepiness Scale (SSS) scores, with higher values reflecting greater subjective sleepiness or lower alertness. Group-level descriptive results by skill level and fatigue status are presented in Supplementary Table S3. Overall, the fatigue group reported markedly higher SSS scores (*M* = 4.44, *SD* = 0.70) than the non-fatigued group (*M* = 1.14, *SD* = 0.49). This difference was statistically significant in the independent-samples t-test (*t* = −23.38, *p* < 0.001, *d* = 6.14), which supports the effectiveness of the mental-fatigue manipulation. In the same vein, the two-way ANOVA showed a significant main effect of fatigue status on SSS scores (*F* = 421.19, *p* < 0.001, ηp^2^ = 0.886). By contrast, neither the main effect of skill level (*F* = 2.443, *p* = 0.095, ηp^2^ = 0.083) nor the interaction between skill level and fatigue status reached significance (*F* = 0.093, *p* = 0.912, ηp^2^ = 0.003). The full ANOVA results are provided in Supplementary Table S4. Taken together, these findings suggest that the manipulation worked in a broadly comparable way across expertise levels, and SSS scores did not differ systematically as a function of skill level. This, in turn, supports the use of the manipulation in the analyses that follow.

### 3.2 Decision accuracy

**Figure 1:**
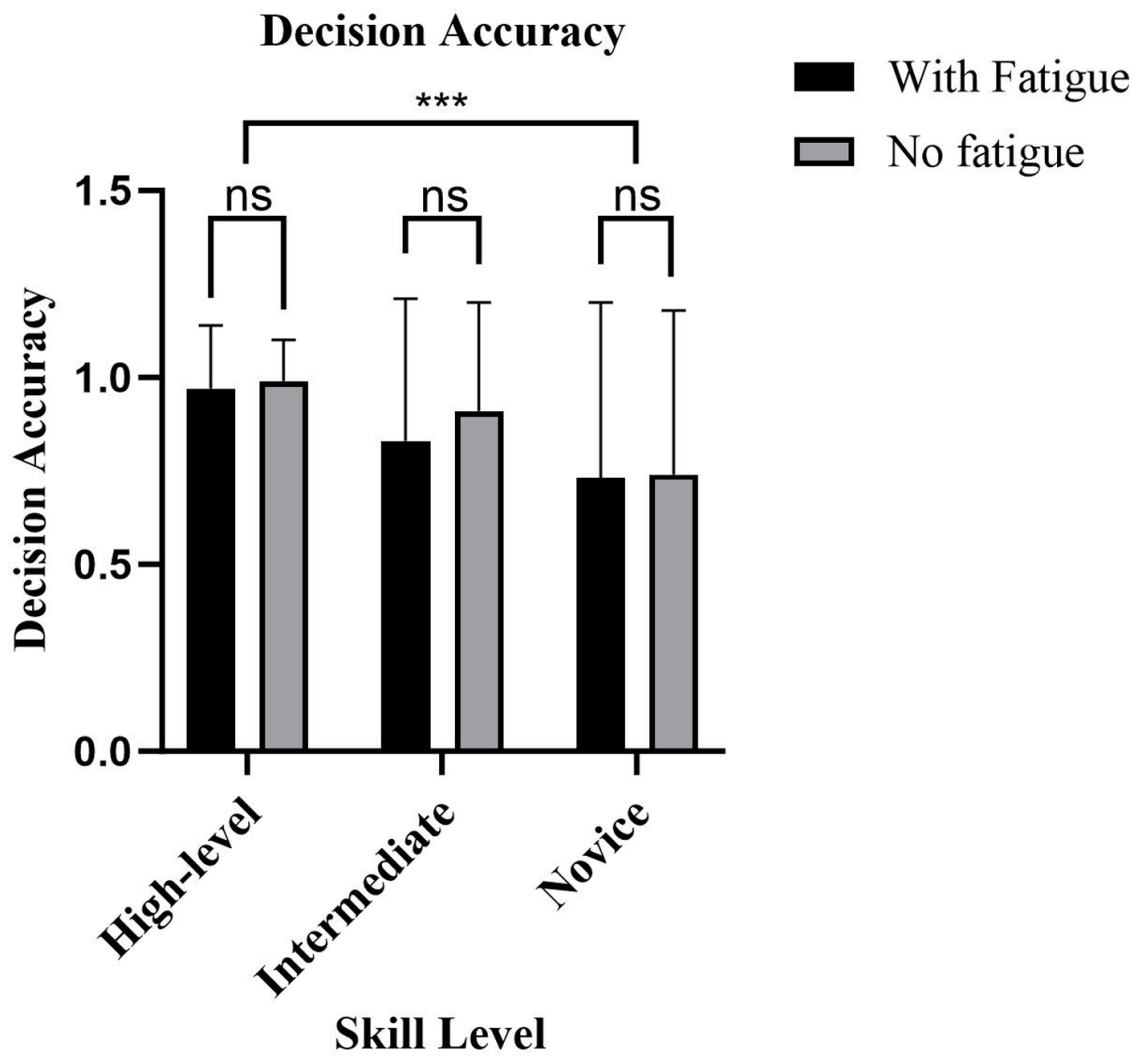
Decision-making accuracy. ***p < 0.001; n.s. = not significant.

Decision accuracy across skill groups and fatigue conditions was first summarized descriptively (Supplementary Table S5), and then analyzed with a 3 (skill level) × 2 (fatigue status) two-way ANOVA. A clear main effect of skill level emerged (*F* = 85.67, *p* < 0.001, ηp^2^ = 0.760), indicating substantial differences in accuracy among the groups regardless of fatigue (Figure 1). *Post hoc* comparisons revealed that the high-skill group (*M* = 0.98) and the intermediate group (*M* = 0.87) both outperformed novices (*M* = 0.73). The main effect of fatigue status, by contrast, was marginal (*F* = 3.64, *p* = 0.057, ηp^2^ = 0.063). Accuracy tended to be lower under fatigue, but this reduction did not reach the conventional significance threshold. The interaction between skill level and fatigue was likewise non-significant (*F* = 0.89, *p* = 0.411, ηp^2^ = 0.032), suggesting that mental fatigue affected accuracy similarly across all expertise levels rather than selectively impairing any particular group.

### 3.3 Decision reaction time

**Figure 2:**
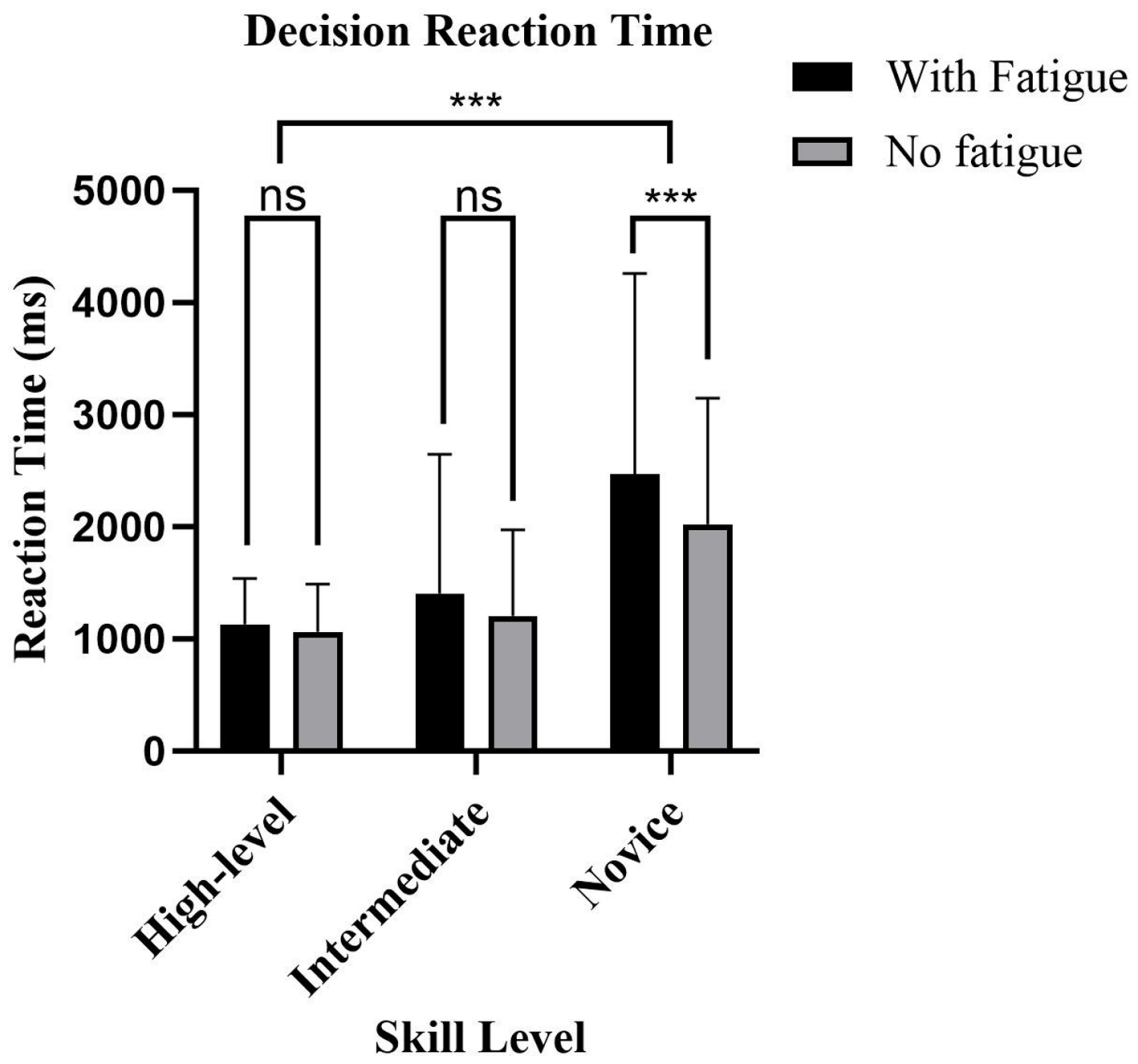
Decision-making reaction time. ***p < 0.001; n.s. = not significant.

Decision reaction time was calculated as the average latency required to respond to each tactical scenario. Descriptive statistics for all groups under both fatigue conditions appear in Supplementary Table S6. A two-way ANOVA revealed a pronounced main effect of fatigue (*F* = 13.73, *p* < 0.001, ηp^2^ = 0.203), with participants in the fatigued condition responding more slowly than those in the non-fatigued condition. Skill level also exerted a clear effect (*F* = 185.15, *p* < 0.001, ηp^2^ = 0.873): high-skill participants were fastest, intermediate players followed, and novices lagged behind. The significant skill × fatigue interaction (*F* = 4.73, *p* = 0.009, ηp^2^ = 0.149) indicates differential effects of fatigue across skill levels. Simple-effects analyses (Figure 2) indicated that novices slowed markedly under fatigue compared to their non-fatigued performance (*p* < 0.001; mean difference = 450 ms). In contrast, neither the high-skill (*p* = 0.524) nor the intermediate group (*p* = 0.167) exhibited significant slowing. Taken together, these findings imply that greater expertise may buffer against fatigue-induced delays in decision-making, while novices appear particularly vulnerable to the same cognitive load.

### 3.4 Eye-tracking measures

The primary eye-tracking outcomes were fixation count, mean fixation duration, and saccade count, supplemented by qualitative visualizations in the form of scanpaths and heatmaps. Considered together, these measures provide a more complete picture of visual-search behavior during task performance.

#### 3.4.1 Fixation count

**Figure 3:**
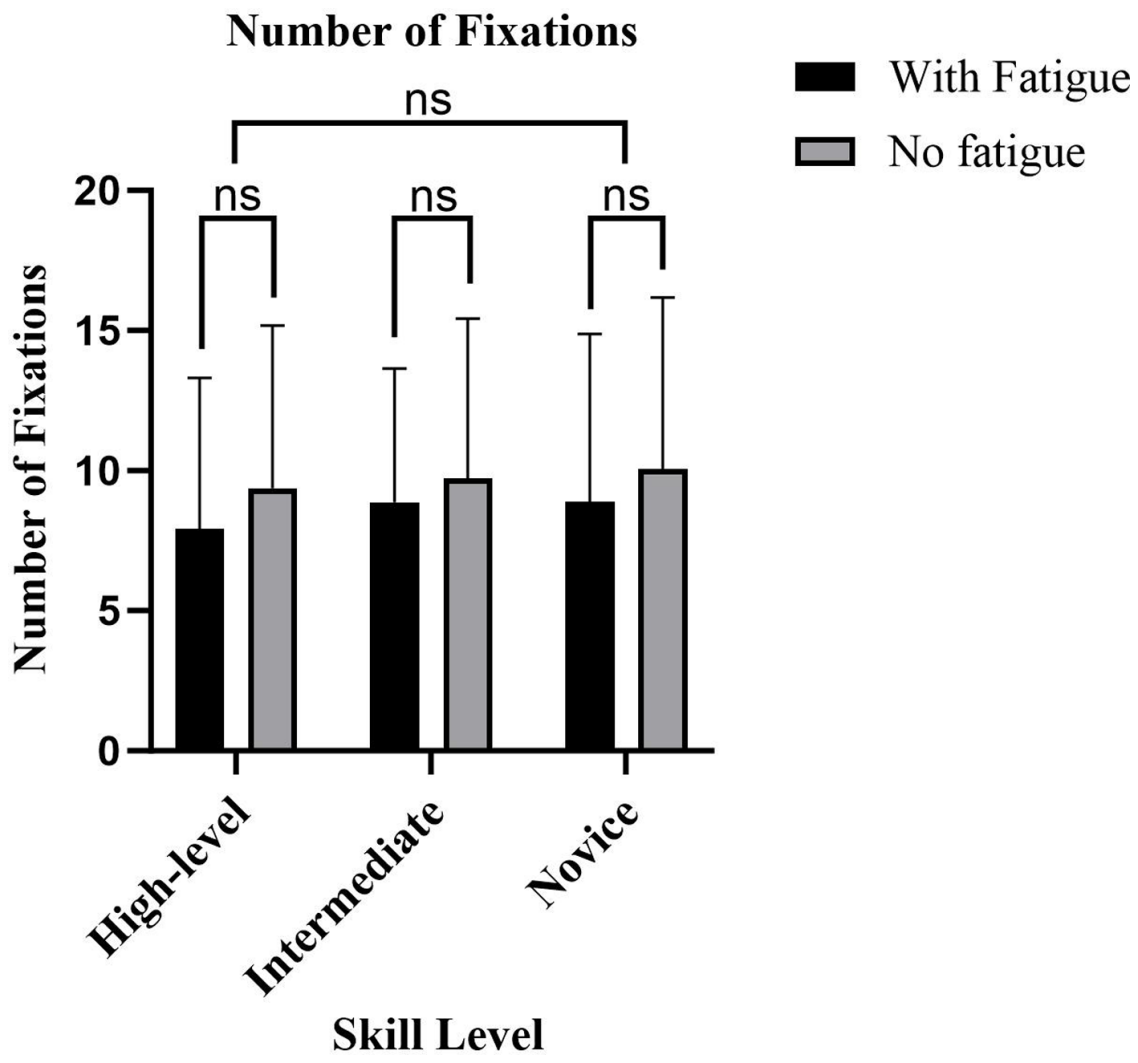
Fixation count. N.s., not significant.

Fixation count was defined as the total number of gaze fixations generated during the decision-making task. Descriptive statistics are presented in Supplementary Table S7. The two-way ANOVA showed no significant main effect of skill level (*F* = 1.25, *p* = 0.286, ηp^2^ = 0.044), suggesting that fixation counts were broadly comparable across the high-skill (*M* = 8.65), intermediate (*M* = 9.30), and novice groups (*M* = 9.50) (see Figure 3). The main effect of fatigue status was likewise not significant (*F* = 1.28, *p* = 0.258, ηp^2^ = 0.023). Although the fatigued condition produced a lower mean fixation count (*M* = 8.56) than the non-fatigued condition (*M* = 9.73), the difference was not statistically reliable. Nor was the interaction between skill level and fatigue status significant (*F* = 0.26, *p* = 0.775, ηp^2^ = 0.010). In other words, neither expertise nor fatigue produced a measurable change in fixation count in the present task.

#### 3.4.2 Fixation duration

**Figure 4:**
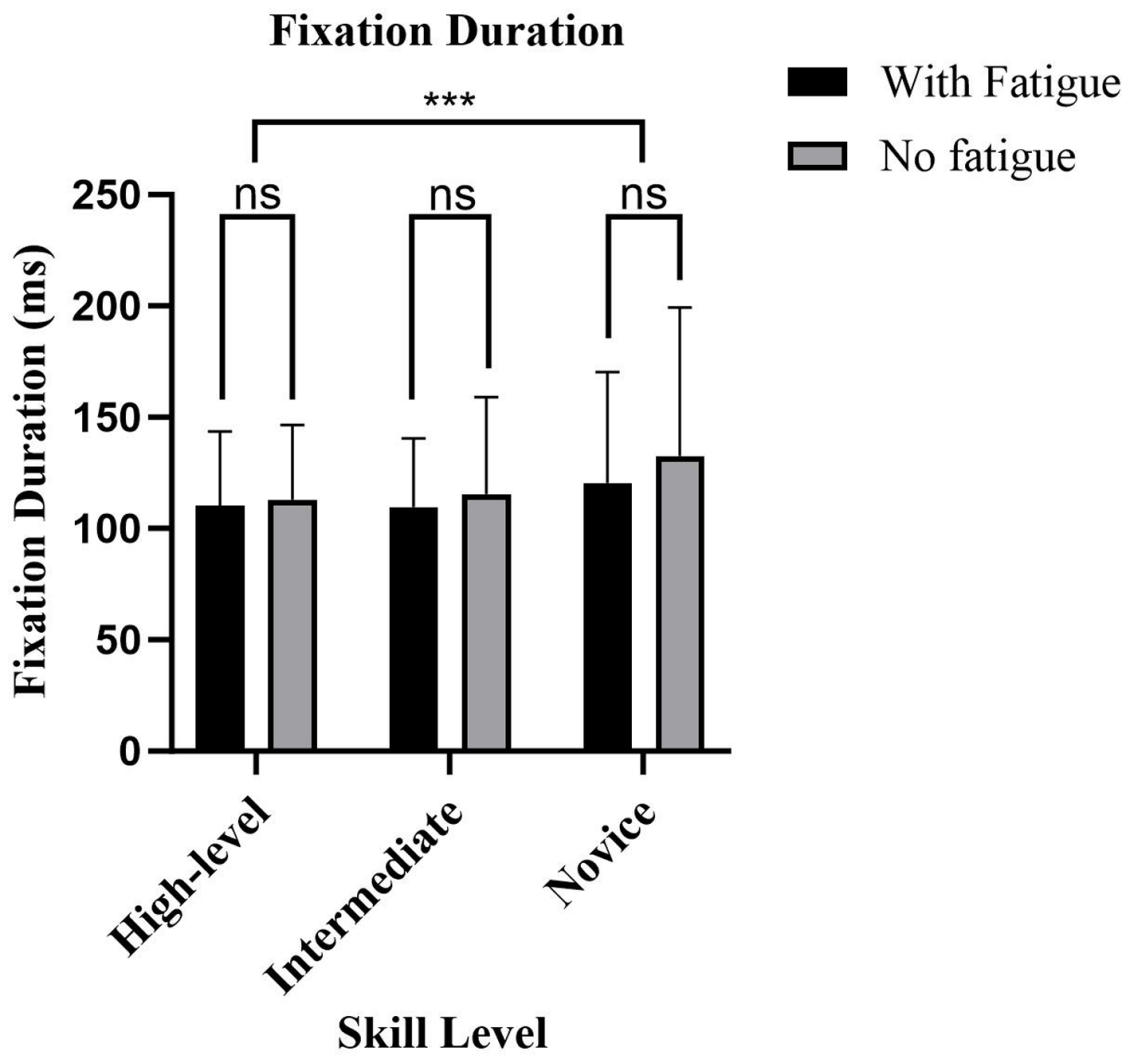
Mean fixation duration. ***p < 0.001; n.s., not significant.

Fixation duration was calculated as the average time, in milliseconds, during which gaze remained relatively stable on a target. This measure is often discussed in relation to the extraction and processing of visual information, although it does not provide a direct index of processing efficiency. Descriptive statistics for all groups are provided in Supplementary Table S8. Analysis via two-way ANOVA revealed a significant main effect of skill level (*F* = 17.00, *p* < 0.001, ηp^2^ = 0.386; Figure 4). Novices exhibited longer fixations (*M* = 127.24 ms) compared to both high-skill (*M* = 111.60 ms) and intermediate participants (*M* = 112.91 ms). This pattern may be consistent with more efficient extraction of task-relevant visual information among more skilled participants, although fixation duration alone cannot establish the underlying processing mechanism. In contrast, fatigue status showed no significant main effect (*F* = 0.86, *p* = 0.353, ηp^2^ = 0.016), nor did the interaction between skill level and fatigue (*F* = 1.71, *p* = 0.181, ηp^2^ = 0.060). These results suggest that group differences in mean fixation duration were more strongly associated with skill level than with the fatigue manipulation used here.

#### 3.4.3 Saccade count

**Figure 5:**
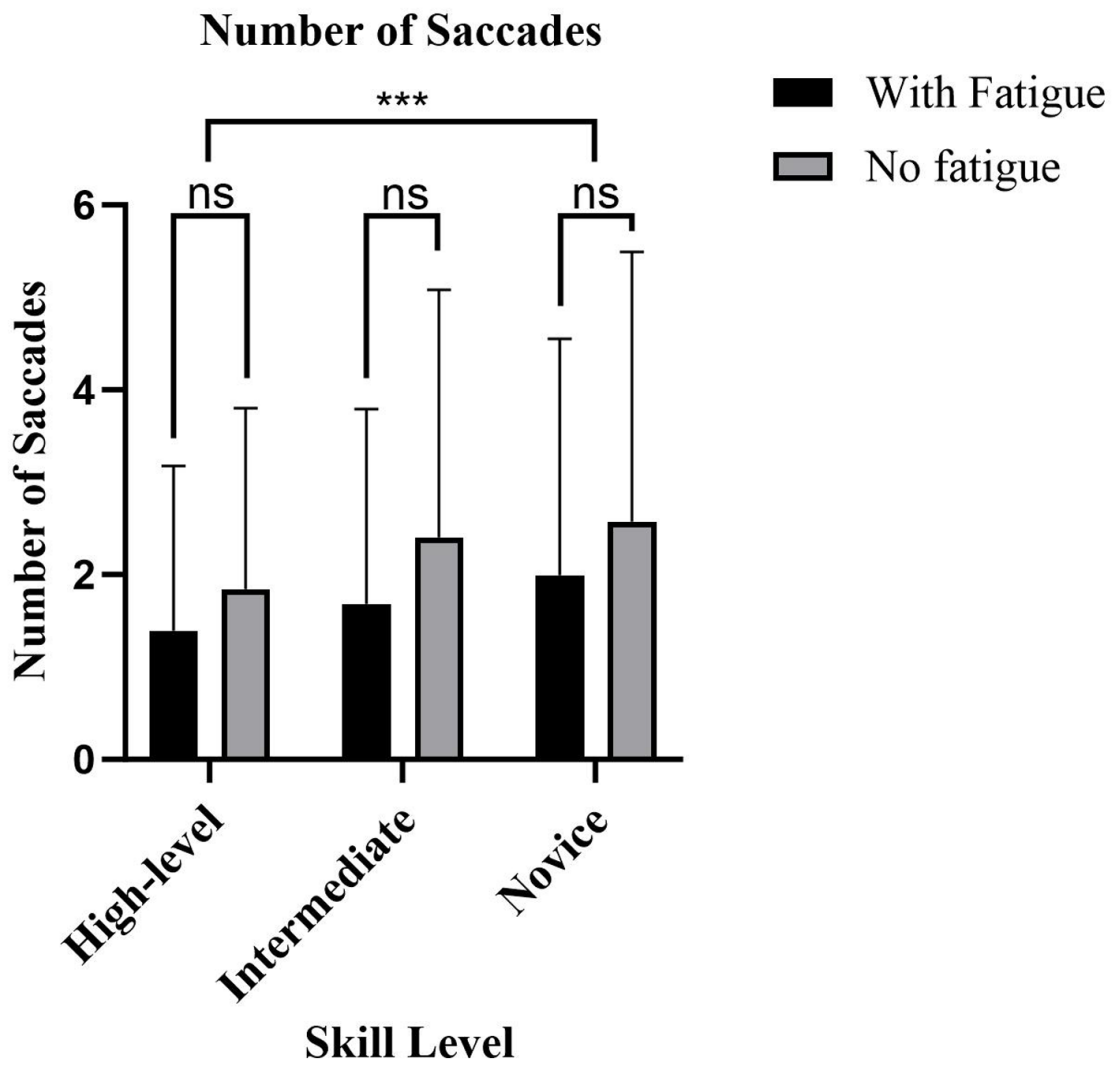
Saccade count. ***p < 0.001; n.s., not significant.

Saccade count referred to the number of rapid eye movements used to shift foveal fixation from one location to another, and it was treated here as a measure related to the breadth and dynamics of visual search. Descriptive statistics are listed in Supplementary Table S9. The two-way ANOVA showed a significant main effect of skill level (*F* = 8.57, *p* < 0.001, ηp^2^ = 0.241). Both the high-skill group (*M* = 1.62) and the intermediate group (*M* = 2.04) made fewer saccades than the novice group (*M* = 2.28), a pattern that may be consistent with more economical and selective search in more skilled participants (see Figure 5). There was also a significant main effect of fatigue status (*F* = 5.35, *p* = 0.021, ηp^2^ = 0.090): participants produced fewer saccades under fatigue (*M* = 1.69) than in the non-fatigued condition (*M* = 2.27). This reduction may reflect a narrowed search scope or a compensatory reduction in exploratory scanning under fatigue. The interaction between skill level and fatigue status was not significant (*F* = 0.30, *p* = 0.740, ηp^2^ = 0.011), indicating that the fatigue-related decrease in saccadic activity was broadly similar across skill levels.

#### 3.4.4 Scanpath visualization

**Figure 6:**
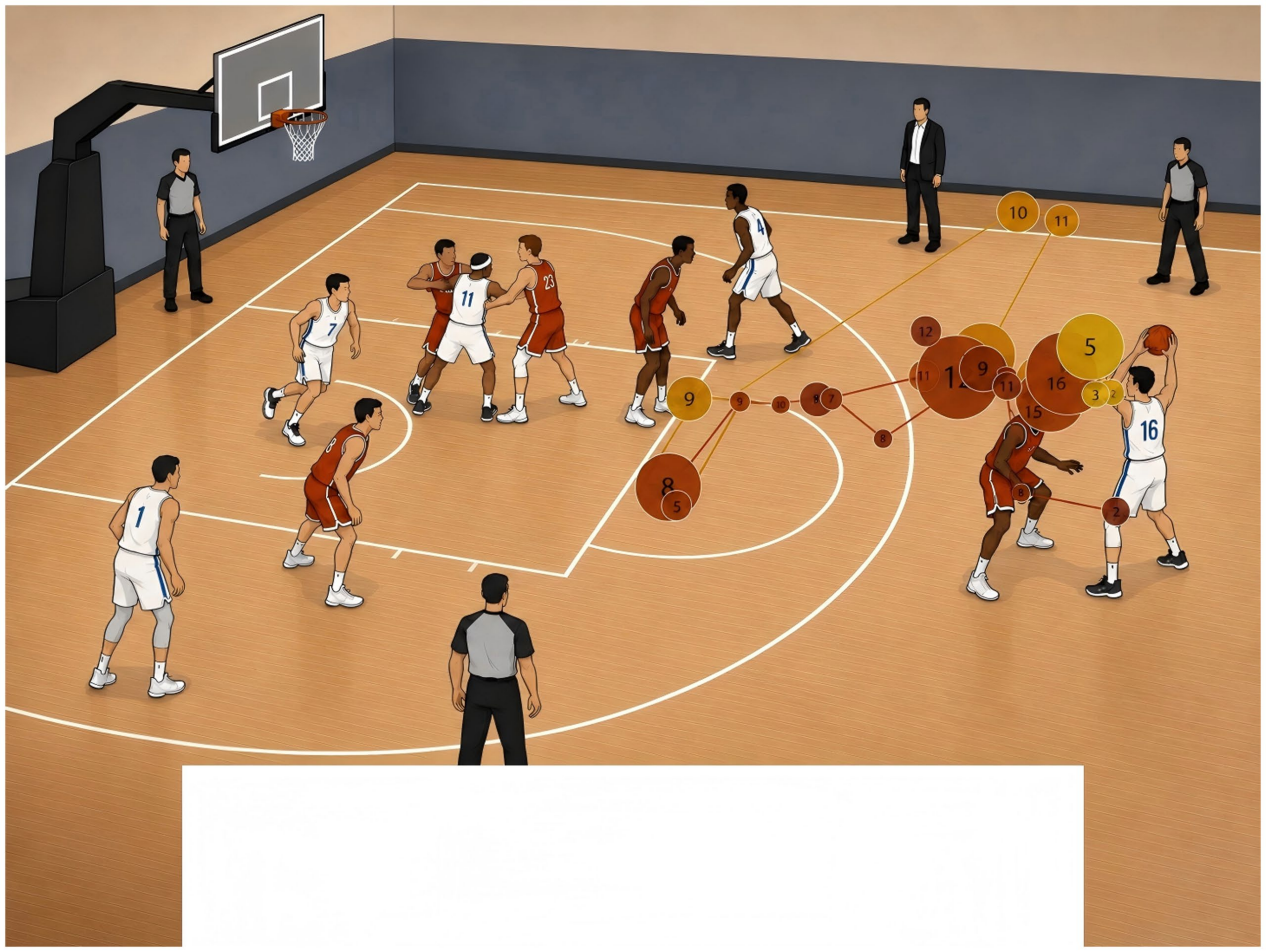
Scanpaths of high-skill basketball players under the non-fatigued condition.

**Figure 7:**
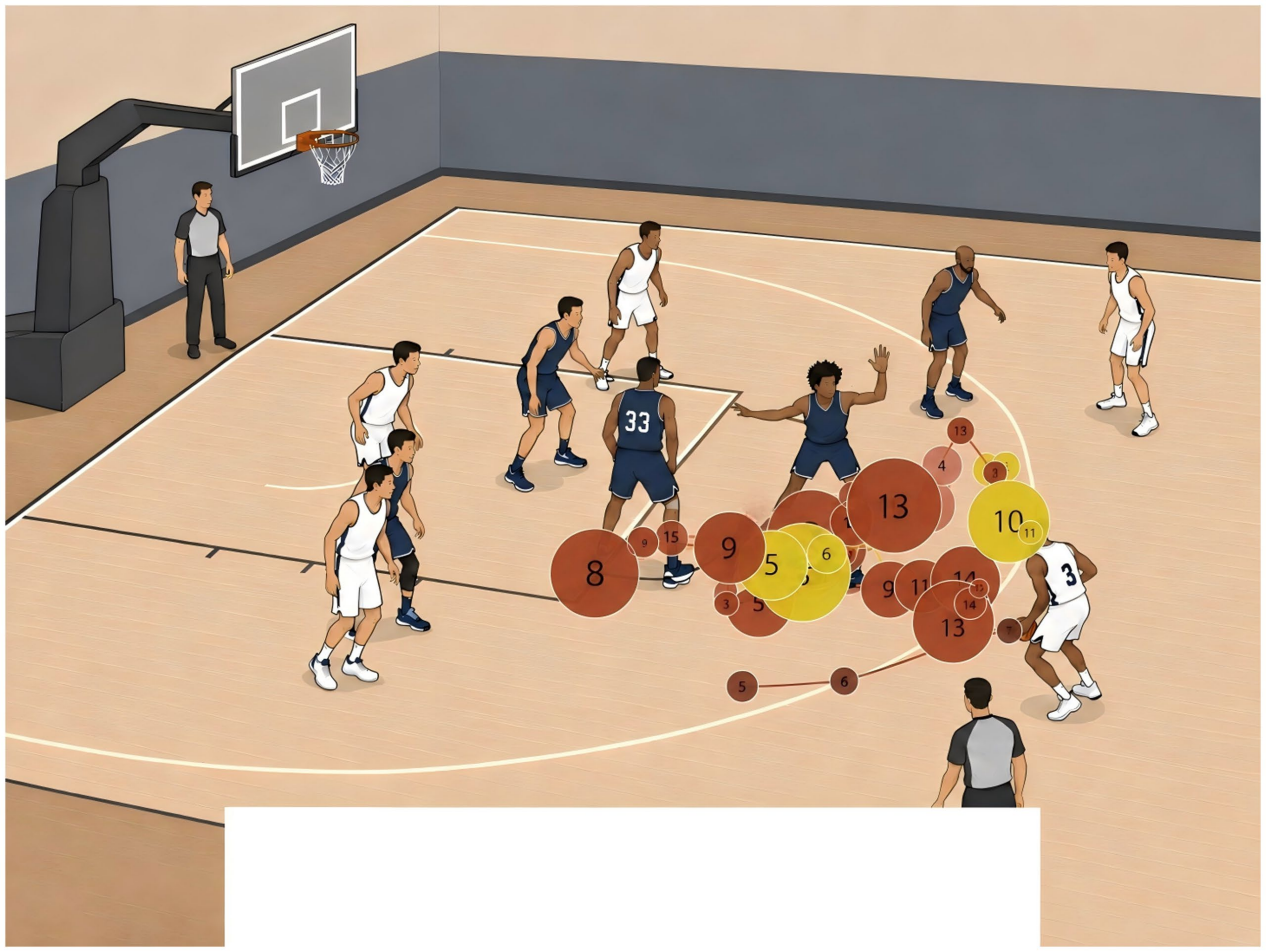
Scanpaths of high-skill basketball players under the fatigued condition.

**Figure 8:**
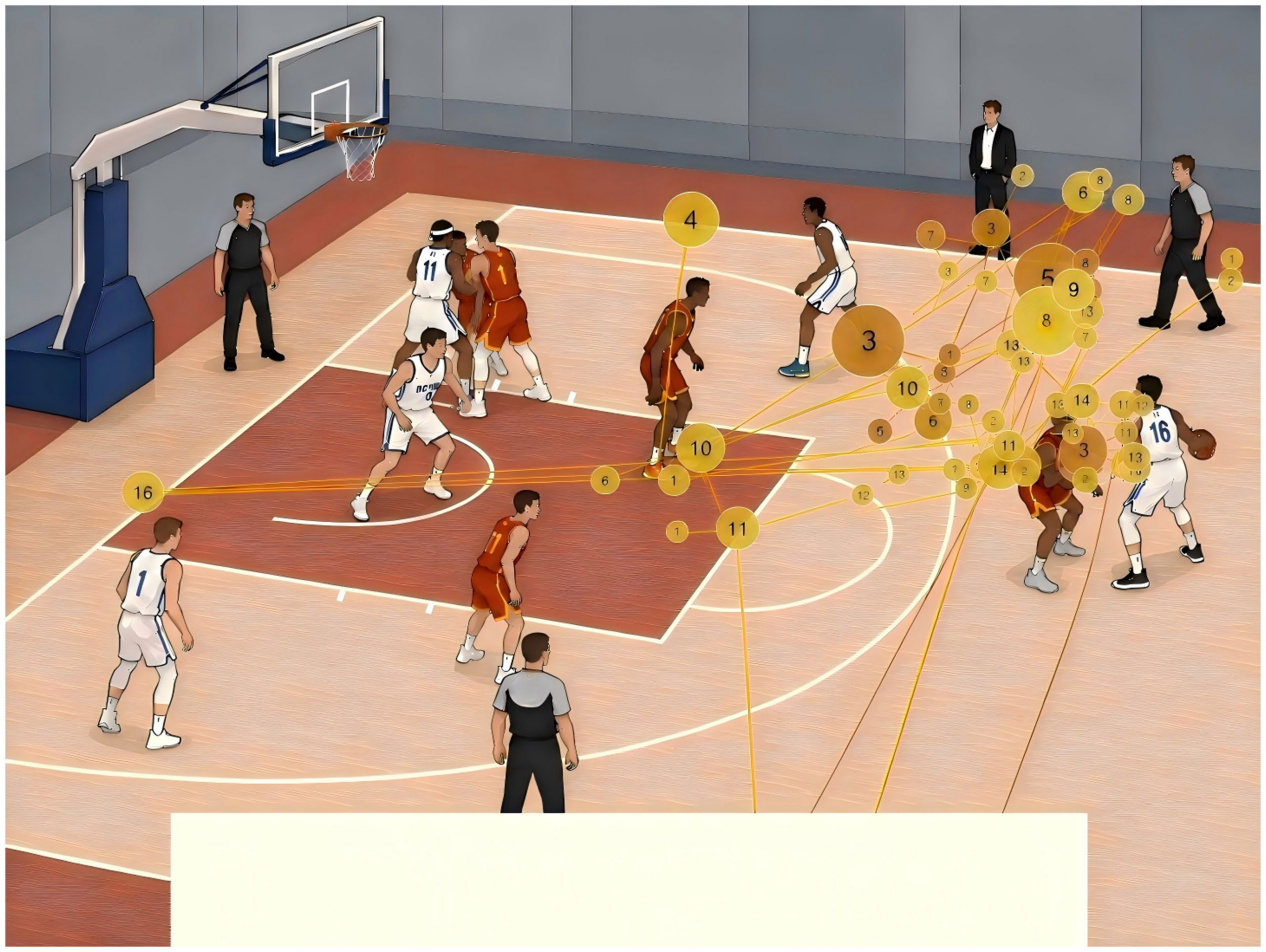
Scanpaths of novice basketball players under the non-fatigued condition.

**Figure 9:**
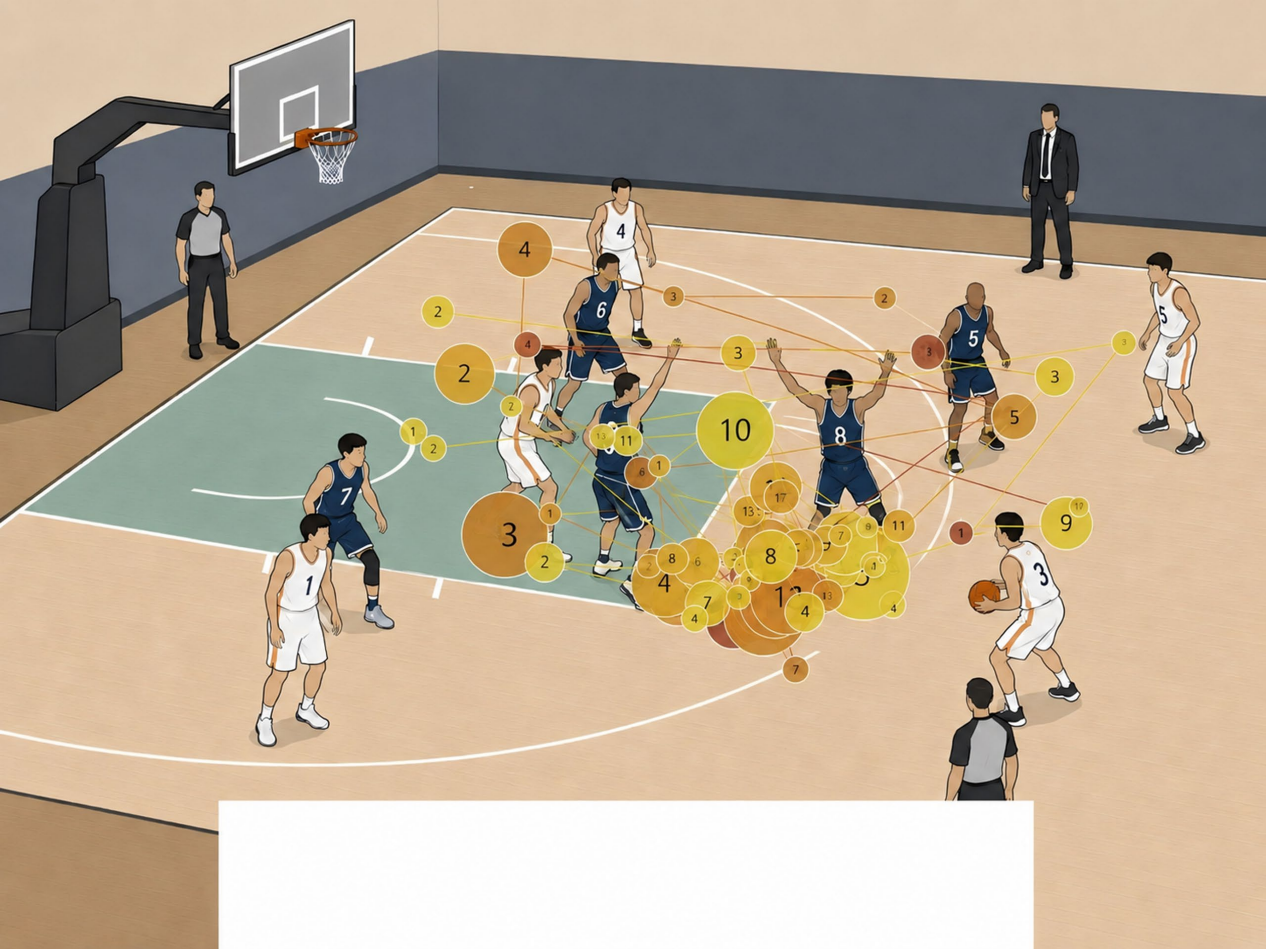
Scanpaths of novice basketball players under the fatigued condition.

The scanpath figures shown below provide descriptive examples drawn from representative trials in the high-skill and novice groups under fatigued and non-fatigued conditions (see Figures 6–9). For each subgroup, the representative trial was defined as the correct trial whose reaction time was closest to the subgroup median. These figures are presented as qualitative illustrations intended to supplement the quantitative analyses and should not be interpreted as inferential evidence.

Across the representative trials, the high-skill group tended to show more spatially concentrated scanpaths than the novice group. In the non-fatigued condition (Figure 6), gaze transitions in the high-skill group appeared to recur among a relatively limited set of scene locations, producing a more compact and visibly ordered pattern. In the corresponding novice example (Figure 8), gaze transitions appeared more widely distributed across the display, with less obvious spatial regularity. These visual differences may be consistent with a more selective search pattern in the high-skill group and a more diffuse one in the novice group, although strong conclusions cannot be drawn from single representative trials.

A similar contrast was visible under fatigue. In the high-skill fatigued example (Figure 7), the overall scanpath configuration remained relatively concentrated and did not appear markedly different from its non-fatigued counterpart. By contrast, the novice fatigued example (Figure 9) still showed dispersed gaze allocation, but with fewer visible transitions across the screen than in the corresponding non-fatigued trial. This pattern did not appear to reflect a shift toward a more organized search strategy; rather, it may be more consistent with reduced overall exploration while maintaining a relatively diffuse search pattern. Broadly considered, the scanpath visualizations suggest that fatigue may have been associated with less extensive visible gaze movement in the novice group, whereas the high-skill group showed less obvious qualitative change in the representative trials.

#### 3.4.5 Heatmap visualization

**Figure 10:**
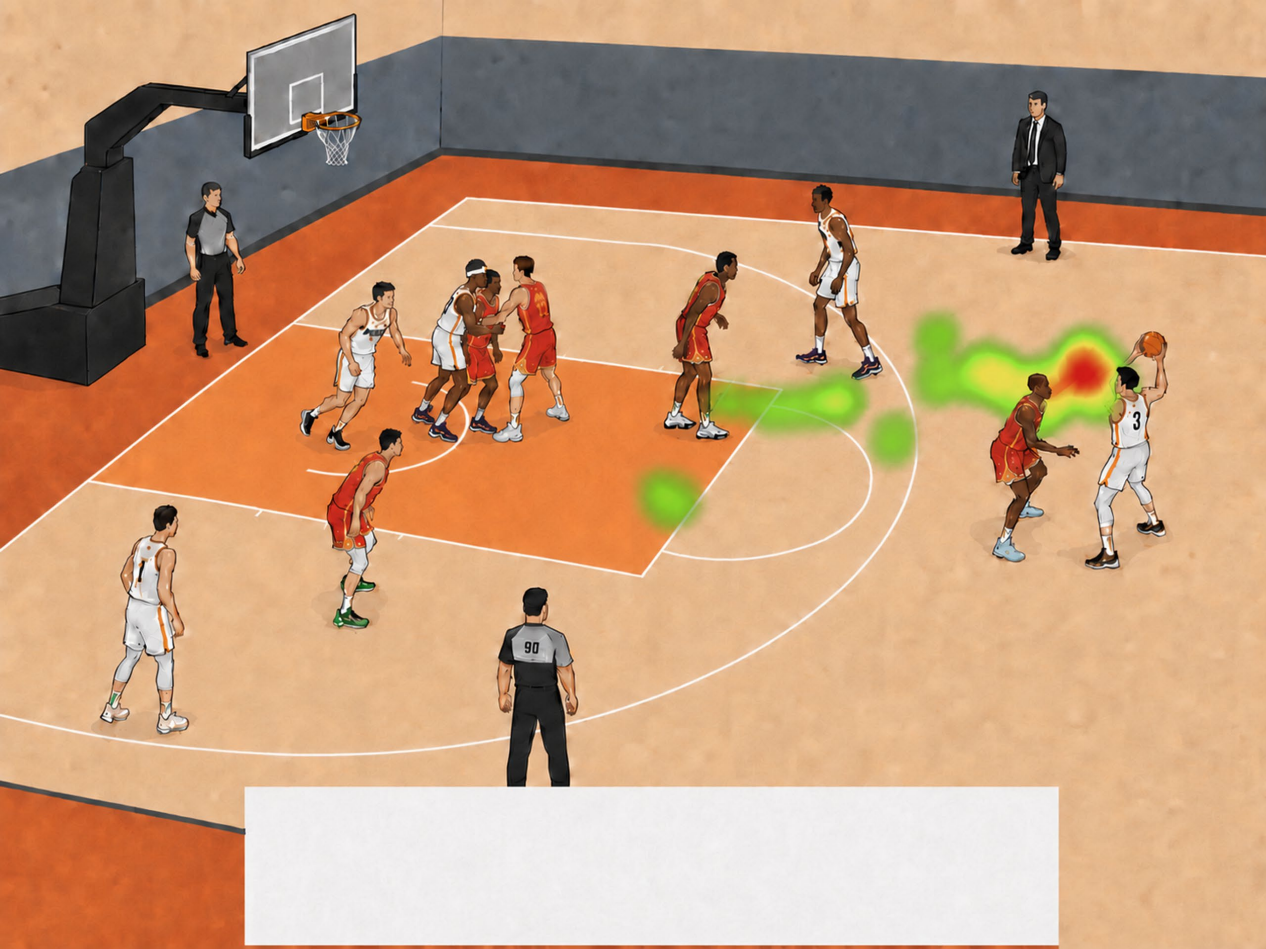
Heatmaps of high-skill basketball players under the non-fatigued condition.

**Figure 11:**
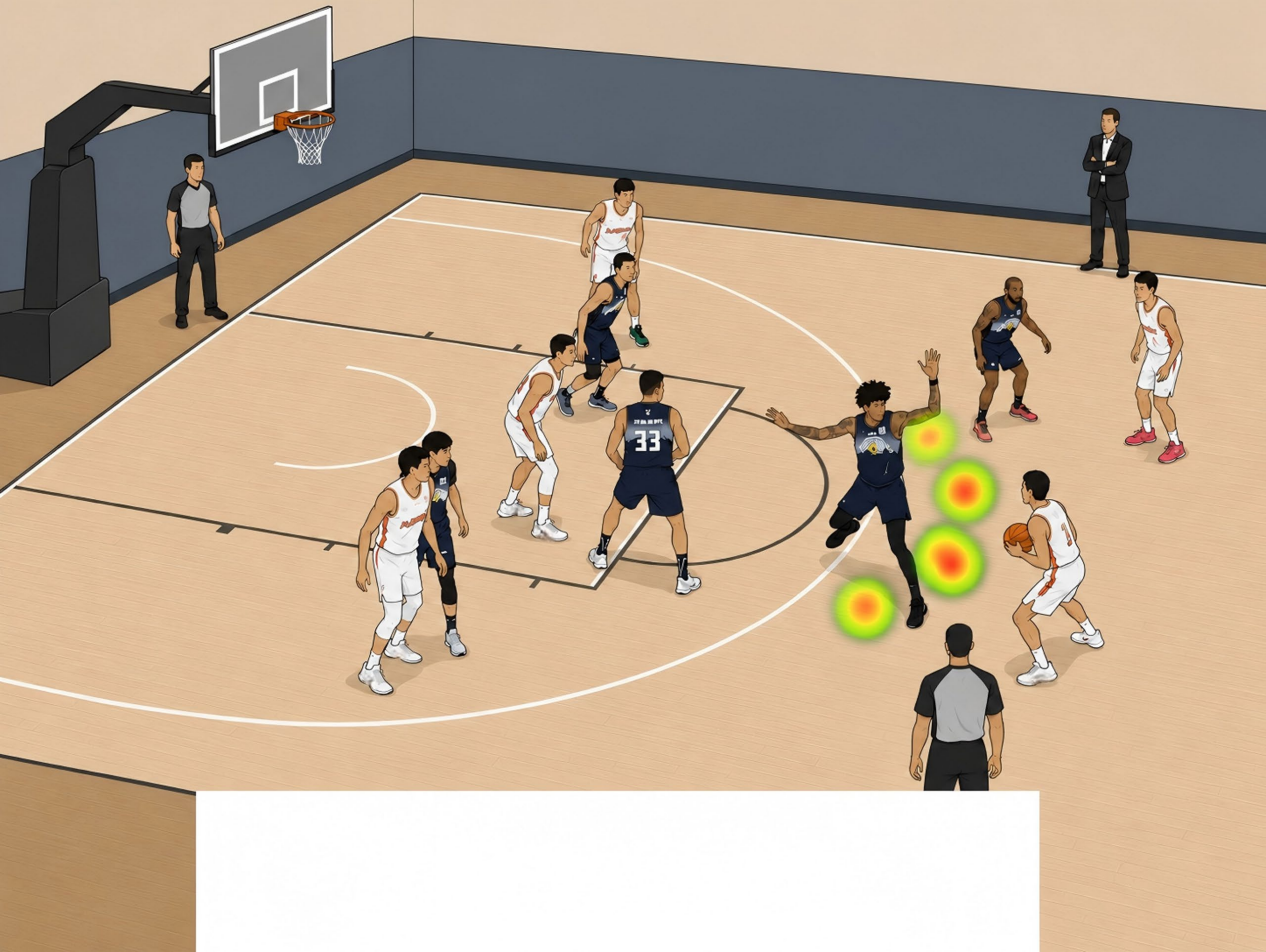
Heatmaps of high-skill basketball players under the fatigued condition.

**Figure 12:**
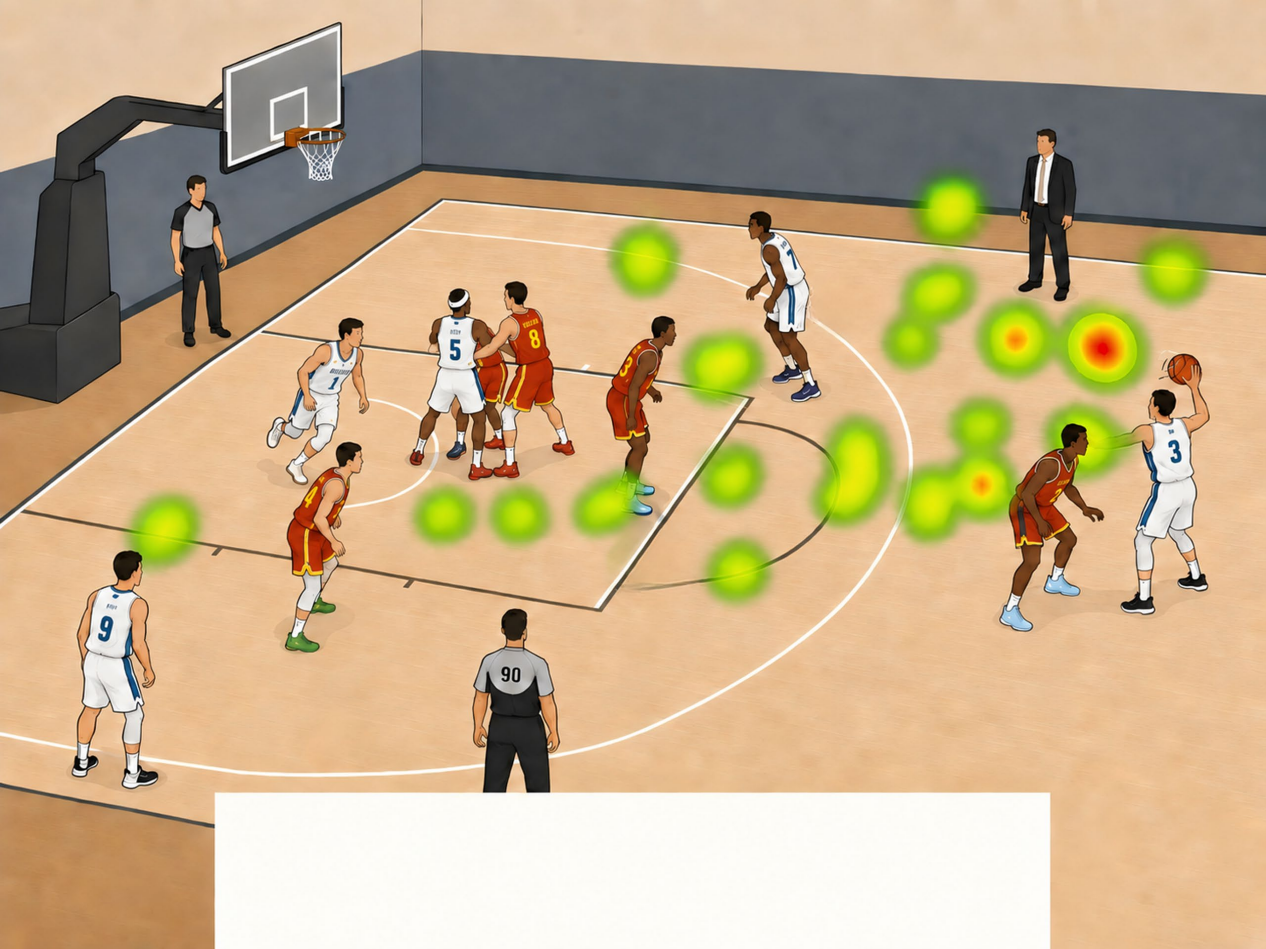
Heatmaps of novice basketball players under the non-fatigued condition.

**Figure 13:**
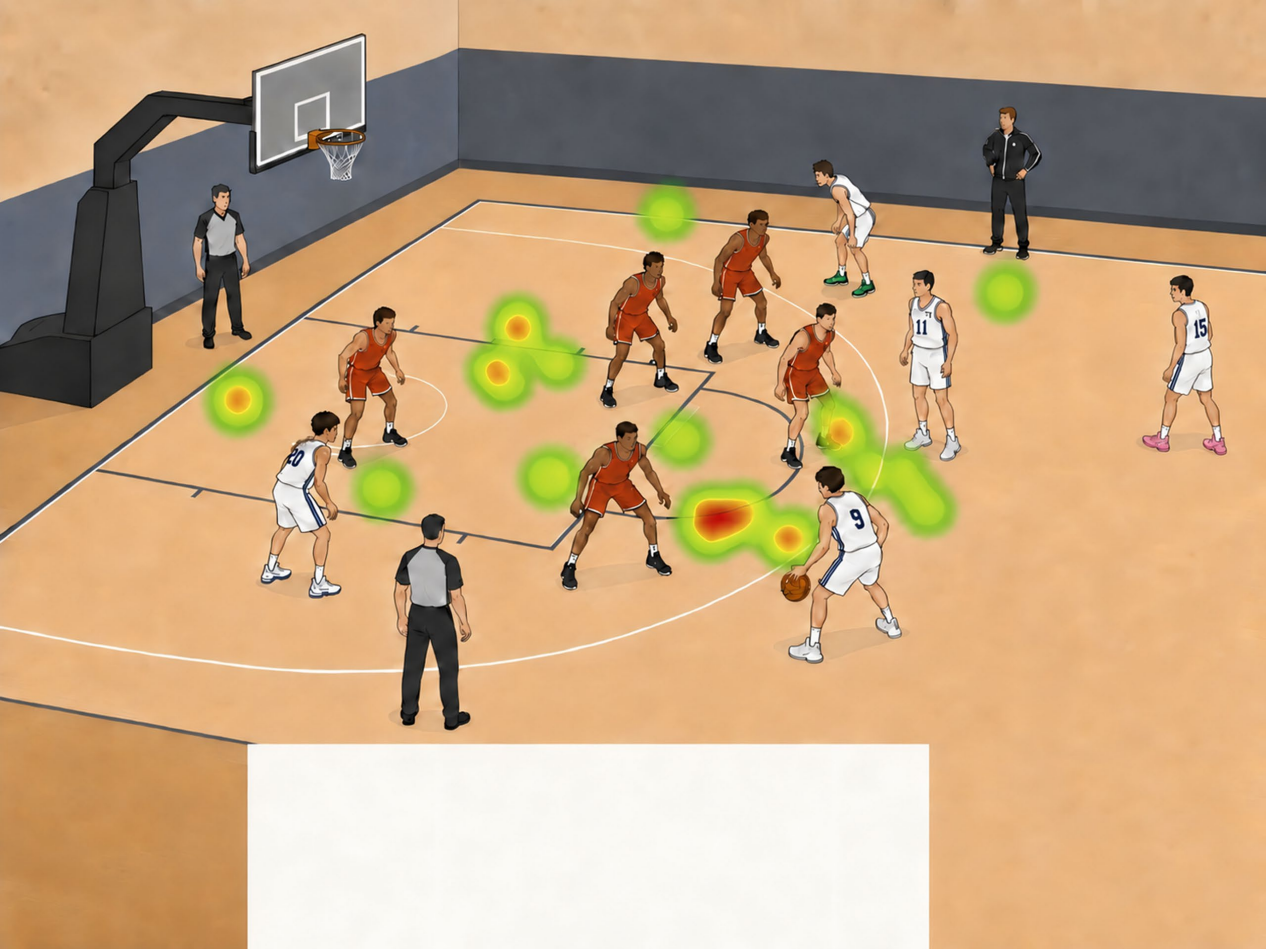
Heatmaps of novice basketball players under the fatigued condition.

The heatmaps provide a descriptive summary of fixation density for the same representative trials used in the scanpath analysis (see Figures 10–13). Warmer colors indicate relatively greater fixation density, whereas cooler colors indicate lower fixation density. As with the scanpath figures, these visualizations are presented to complement the quantitative findings and should be interpreted cautiously.

The representative heatmaps from the high-skill group appeared more spatially concentrated than those from the novice group. In the non-fatigued high-skill example (Figure 10), fixation density was distributed across two relatively focal regions, suggesting repeated allocation of gaze to a limited number of areas within the scene. In the corresponding novice example (Figure 12), fixation density appeared more diffuse, without a similarly clear concentration pattern. This visual contrast broadly aligns with the scanpath observations and may be consistent with more selective spatial allocation in the high-skill example.

Under fatigue, the overall heatmap structure in the high-skill example (Figure 11) remained broadly similar to that observed in the non-fatigued condition, with no obvious large-scale redistribution of fixation density. In the novice fatigued example (Figure 13), fixation density still appeared relatively diffuse, but the overall intensity of the heatmap seemed lower than in the corresponding non-fatigued trial. Rather than suggesting greater spatial concentration, this pattern may be more consistent with reduced overall fixation density while preserving a relatively broad distribution. Taken together, the heatmap visualizations may be consistent with reduced visual exploration under fatigue in the novice group, whereas the representative high-skill trials showed less visible qualitative change.

## 4 Discussion

This section interprets changes in basketball-specific decision-making performance and visual search behavior among participants with different skill levels under fatigue. Before discussing the main behavioral and eye-tracking findings, it is important to note that participants reported higher SSS scores in the fatigue condition. This result indicates increased subjective sleepiness or reduced alertness after the induction task and supports the effectiveness of the manipulation check. Because the SSS primarily reflects momentary sleepiness and alertness, the effects of mental fatigue are interpreted in relation to changes in response time and eye-movement measures. The following sections discuss skill-level differences in decision-making performance and visual search, the behavioral consequences of mental fatigue, the potential moderating role of skill level, and changes in gaze transition measures under fatigue. Finally, the study limitations and directions for future research are addressed.

### 4.1 Sport-specific experience, decision-making performance, and visual search patterns

The present study showed that skill level was closely associated with accuracy and response time in the basketball video-based decision-making task. High-level participants achieved higher decision-making accuracy and shorter response times, followed by intermediate-level participants, whereas novices showed relatively poorer performance. This progressive pattern is consistent with expert–novice research, which suggests that long-term training and competition experience promote the development of sport-specific knowledge structures, tactical representations, and cue-utilization abilities. These capacities enable athletes to identify key information, evaluate on-court relationships, and select appropriate responses more effectively in dynamic situations (Mann et al., 2007; Williams et al., 2004; Vater, 2024). By contrast, novices may not yet have developed stable situational recognition or cue-prioritization abilities. Consequently, when confronted with information-dense basketball scenarios, they may require more time to filter relevant information and confirm decisions (Bossard et al., 2022; Wickemeyer et al., 2024).

The eye-tracking results further indicated that skill-related differences were primarily reflected in mean fixation duration and saccade count, rather than fixation count. The longer mean fixation duration and higher saccade count observed in novices may indicate greater processing demands during the extraction, confirmation, and integration of scene information. In contrast, the shorter fixation durations and fewer saccades observed in high- and intermediate-level participants may reflect more stable information selection and reduced need for repeated confirmation (Abernethy, 1988; Silva et al., 2022; Van Maarseveen et al., 2018). Thus, participants with different skill levels differed not only in decision-making performance but also in how they organized visual information.

### 4.2 Mental fatigue and slowed decision responses

In the present study, the fatigue induction condition was associated with prolonged response time in the basketball video-based decision-making task, whereas accuracy did not decrease significantly. This pattern is consistent with previous sport-related studies showing that mental fatigue can increase response time during soccer- and basketball-specific decision-making tasks (Smith et al., 2016; Fortes et al., 2022). It may also reflect reduced attentional control and slower response preparation under cognitive fatigue, thereby increasing the time required for cue selection, situational judgment, and response selection (Boksem and Tops, 2008; Hopstaken et al., 2016). Because the task did not impose a strict external time limit, participants may have maintained decision quality by taking longer to respond. This finding aligns with the speed–accuracy trade-off framework, which suggests that individuals may slow their responses to preserve task success (Langner et al., 2010; Rozand et al., 2015).

The absence of a significant decline in accuracy does not imply that mental fatigue has a limited effect on basketball decision-making. Under the present task conditions, its influence was mainly expressed as delayed decision responses. In basketball, passing windows, driving opportunities, defensive rotations, and help-defense decisions are often highly time constrained. Even small delays may therefore reduce the timeliness and effectiveness of actions in real-game situations (Kinrade et al., 2015; Kostrna, 2022; Guo and Wang, 2025). Future studies should use on-court tasks or more interactive experimental paradigms to examine how response delays under mental fatigue translate into changes in actual game performance.

### 4.3 Potential moderating role of skill level in fatigue-related response delays

The present study found an interaction between skill level and fatigue state for response time. Specifically, novices showed a greater increase in response time under fatigue, whereas changes were smaller among high- and intermediate-level participants. This finding suggests that mental fatigue does not affect basketball decision-making speed uniformly across participants. Instead, its effects may depend on sport-specific experience and associated information-processing strategies. Higher-level athletes typically have more stable perceptual–cognitive representations, stronger cue-prioritization abilities, and greater situational recognition experience. When cognitive resources are challenged, these characteristics may help higher-level athletes maintain decision-making efficiency to some extent by relying on more practiced or schematic judgment processes (Belling et al., 2015; Moran et al., 2019).

By contrast, novices may depend more on broad information search and comparisons among multiple response options. When mental fatigue reduces available cognitive resources, this search- and comparison-based decision-making strategy may be more vulnerable to disruption, resulting in longer response times (Van Der Linden et al., 2003; Fortes et al., 2022). This interpretation is consistent with previous research suggesting that the effects of mental fatigue on open-skill sport performance are not fixed, but vary according to sport context, task demands, and athlete characteristics (Coyne et al., 2021; Pan et al., 2025). However, because the present study did not directly measure specific cognitive processing mechanisms, the findings should not be interpreted as evidence that high-level athletes possess a distinct resistance mechanism against mental fatigue. Thus, skill level may influence how mental fatigue affects basketball decision-making speed. Training for less experienced athletes may therefore benefit from greater emphasis on fatigue management, situational recognition, and rapid decision-making to reduce the risk of delayed responses under fatigue.

### 4.4 Changes in gaze transition indicators under fatigue

In the present study, mental fatigue did not affect all eye-movement indicators consistently. Fatigue-related differences were primarily observed in saccade count, whereas fixation count and mean fixation duration did not change significantly. This finding suggests that, in the current basketball video-based decision-making task, mental fatigue may be more evident in gaze transition activity than in stable fixation processes. Previous studies have similarly shown that eye-movement control indicators, including saccade velocity, the peak velocity–amplitude relationship, microsaccades, and ocular drift, are sensitive to fatigue states (Bafna and Hansen, 2021; Di Stasi et al., 2013). Thus, the observed change in saccade count may provide preliminary evidence of altered gaze transition activity under fatigue. However, because the present study did not examine dynamic saccade measures, such as velocity, amplitude, or latency, the specific eye-movement control mechanisms underlying this change remain unclear.

The reduction in saccade count should be interpreted cautiously. It may indicate that participants made fewer gaze shifts between information areas under fatigue, resulting in a narrower range of visual exploration and reduced scene updating. Alternatively, it may reflect an adaptive adjustment of visual exploration when cognitive resources were limited, reducing the processing load associated with frequent attentional shifts (Hockey, 1997). In basketball, key information is typically distributed across teammates, opponents, ball-possession changes, and spatial relationships. Fewer gaze transitions may therefore impair the continuous monitoring of dynamic tactical information. However, this interpretation requires further examination using more comprehensive eye-movement indicators and detailed temporal analyses (Klostermann and Moeinirad, 2020).

The scan path and heat map results provided descriptive spatial support for the quantitative eye-movement findings. In particular, the scan paths helped illustrate changes in gaze transitions between key areas, whereas the heat maps showed whether gaze distribution became more concentrated or constricted under fatigue. However, because these visualizations were based on representative trials, they cannot replace overall statistical analyses. Their interpretation should therefore remain secondary to the quantitative eye-movement results (Kredel et al., 2023). The quantitative eye-movement results, together with the supplementary visualizations, suggest that the fatigue induction condition may be associated with altered gaze transition activity during basketball decision-making tasks. Future studies should use more detailed temporal analyses of eye movements to clarify how such changes affect tactical information extraction and situational updating.

### 4.5 Limitations

Several limitations should be considered when interpreting the findings of this study:

First, this study used a between-group fatigue–control design rather than requiring the same participants to complete both fatigue and non-fatigue conditions. Although this design reduces order effects, learning effects, and residual fatigue effects, it limits the control of individual differences, such as baseline response speed, fatigue susceptibility, and gaze behavior. In addition, the sample included only male university students, and the number of participants in each skill level × fatigue condition cell was relatively small. Because participants were grouped by basketball skill level rather than recruited through fully random sampling, the generalizability of the findings may be limited. In addition, the modest sample size, particularly the relatively small number of participants in each skill × fatigue cell, may have reduced the statistical power to detect smaller effects. Therefore, non-significant interaction effects and small differences in eye-movement measures should be interpreted cautiously.

Second, this study used a Stroop-type cognitive task to induce mental fatigue. The Stroop task is commonly used in mental fatigue research because it involves sustained attention, conflict monitoring, and inhibitory control. However, because no alternative fatigue induction methods were compared, it remains unclear whether the present findings generalize to other cognitive tasks or sport-specific fatigue protocols. The SSS was used as a manipulation check because it reflects changes in subjective sleepiness or alertness. However, sleepiness and mental fatigue are related but distinct constructs. Future studies should combine more direct measures of mental fatigue, such as a Visual Analogue Scale, with motivation ratings, cognitive performance checks, or physiological indicators to confirm the fatigue state more comprehensively.

Third, although the basketball video-based decision-making task is more sport-specific than static laboratory tasks, it remains screen based. It therefore cannot fully reproduce the perception–action coupling, interpersonal interactions, physical constraints, and continuous time pressure of real games. Future studies should use immersive simulations, interactive tasks, small-sided games, or on-court decision-making tests to examine how fatigue-related response delays occur in real basketball contexts.

Finally, the eye-tracking analysis was mainly based on global measures, including fixation count, mean fixation duration, and saccade count. These measures provide preliminary evidence but cannot fully determine which information areas participants attended to, how gaze shifted between information sources, or when these changes occurred during the decision-making process. Future research should incorporate areas-of-interest analysis, gaze transition measures, time-series eye-movement analysis, and dynamic saccade indicators, such as amplitude, velocity, and latency. These approaches would help clarify how mental fatigue affects visual search during basketball decision-making.

## 5 Conclusion

This study examined the impact of mental fatigue on basketball-specific tactical decision-making and associated visual-search behavior across different skill levels. Expertise significantly influenced performance outcomes: high-skill and intermediate players responded faster and more accurately than novices. Fatigue, in contrast, primarily slowed responses rather than altering overall accuracy, and this effect was most pronounced among novice participants, suggesting that vulnerability to fatigue-related slowing may vary with skill level.

The eye-tracking results further suggested that fatigue-related effects were more evident in saccadic activity than in fixation count or mean fixation duration. Saccade count declined under fatigue, whereas fixation count and mean fixation duration remained largely stable. When considered together with the qualitative scanpath and heatmap observations, these findings may be consistent with reduced visual exploration under fatigue, particularly among less experienced players. However, these interpretations should be made cautiously, as the present eye-tracking measures do not permit direct conclusions about the underlying cognitive mechanisms.

From a practical perspective, the findings highlight the importance of monitoring not only decision accuracy but also decision speed. Training approaches that help athletes maintain functional visual-search behavior and timely decision-making under mentally fatiguing conditions may be especially beneficial for developing players.

## Data Availability

The original contributions presented in the study are included in the article/Supplementary material, further inquiries can be directed to the corresponding author.
