# Supplementary material for: Effects of mental fatigue on basketball decision-making and visual search behavior
Source: Front Psychol. 2026 Jul 27;17:1837100. doi: 10.3389/fpsyg.2026.1837100 (PMC13453791; doi:10.3389/fpsyg.2026.1837100)
Supplement: Supplementary file 1 [file Table_1.doc]

Table S1. Participant characteristics

| Group | Number of Participants | Athletic Level | Training Experience (Years) | Age |
| --- | --- | --- | --- | --- |
| High-skill group | 20 | National First-Class or Second-Class Athletes | 8.29 ± 1.86 | 20.00 ± 0.88 |
| Intermediate group | 20 | Regularly trained university team players without national athlete certification | 6.83 ± 1.40 | 20.50 ± 0.90 |
| Novice group | 20 | Students without formal basketball training or skill rating | 0.58 ± 1.73 | 20.17 ± 0.72 |

Table S2. Stanford Sleepiness Scale descriptors

| Item | Description |
| --- | --- |
| 1. Feeling active, vital, alert, or wide awake |  |
| 2. Functioning at high levels, but not at peak; able to concentrate |  |
| 3. Awake, but relaxed; responsive but not fully alert |  |
| 4. Somewhat foggy, let down |  |
| 5. Foggy; losing interest in remaining awake; slowed down |  |
| 6. Sleepy, woozy, fighting sleep; prefer to lie down |  |
| 7. No longer fighting sleep, sleep onset soon; having dream-like thoughts |  |

Table S3. Descriptive statistics for Stanford Sleepiness Scale scores by skill level and fatigue status

| Group | No fatigue | With fatigue |
| --- | --- | --- |
| High-skill group | 1.00 ± 0.00 | 4.31 ± 0.48 |
| Intermediate group | 1.00 ± 0.00 | 4.50 ± 0.84 |
| Novice group | 1.31 ± 0.60 | 4.69 ± 0.79 |

Table S4. Two-way ANOVA results for Stanford Sleepiness Scale scores by skill level and fatigue status

| Source | df | F | p | η²ₚ |
| --- | --- | --- | --- | --- |
| Fatigue status | 1 | 421.186 | 0.000*** | 0.886 |
| Skill level | 2 | 2.443 | 0.095 | 0.083 |
| Skill level  ×  Fatigue status | 2 | 0.093 | 0.912 | 0.003 |

Table S5. Descriptive statistics for decision-making accuracy by skill level and fatigue status (M ± SD)

| Group / Fatigue status | No fatigue | With fatigue |
| --- | --- | --- |
| High-skill group | 0.99 ± 0.11 | 0.97 ± 0.17 |
| Intermediate group | 0.91 ± 0.29 | 0.83 ± 0.38 |
| Novice group | 0.74 ± 0.44 | 0.73 ± 0.47 |

Table S6. Descriptive statistics for decision-making reaction time by skill level and fatigue status (M ± SD)

| Group / Fatigue status | No fatigue (ms) | With fatigue (ms) |
| --- | --- | --- |
| High-skill group | 1063.83 ± 424.87 | 1130.73 ± 408.36 |
| Intermediate group | 1205.27 ± 766.99 | 1404.47 ± 1242.73 |
| Novice group | 2023.47 ± 1124.15 | 2473.50 ± 1787.67 |

Table S7. Descriptive statistics for fixation count by skill level and fatigue status (M ± SD)

| Group / Fatigue status | No fatigue (count) | With fatigue (count) |
| --- | --- | --- |
| High-skill group | 9.37 ± 5.81 | 7.92 ± 5.39 |
| Intermediate group | 9.72 ± 5.71 | 8.88 ± 4.76 |
| Novice group | 10.05 ± 6.13 | 8.89 ± 5.98 |

Table S8. Descriptive statistics for mean fixation duration by skill level and fatigue status (M ± SD)

| Group / Fatigue status | No fatigue (ms) | With fatigue (ms) |
| --- | --- | --- |
| High-skill group | 113.04 ± 33.52 | 110.35 ± 33.38 |
| Intermediate group | 115.50 ± 43.53 | 109.76 ± 30.81 |
| Novice group | 132.70 ± 66.51 | 120.51 ± 49.88 |

Table S9. Descriptive statistics for saccade count by skill level and fatigue status (M ± SD)

| Group / Fatigue status | No fatigue (count) | With fatigue (count) |
| --- | --- | --- |
| High-skill group | 1.84 ± 1.96 | 1.39 ± 1.79 |
| Intermediate group | 2.40 ± 2.68 | 1.68 ± 2.11 |
| Novice group | 2.57 ± 2.92 | 1.99 ± 2.56 |
